# A machine learning approach to identify functional biomarkers in human prefrontal cortex for individuals with traumatic brain injury using functional near‐infrared spectroscopy

**DOI:** 10.1002/brb3.541

**Published:** 2016-08-24

**Authors:** Nader Karamzadeh, Franck Amyot, Kimbra Kenney, Afrouz Anderson, Fatima Chowdhry, Hadis Dashtestani, Eric M. Wassermann, Victor Chernomordik, Claude Boccara, Edward Wegman, Ramon Diaz‐Arrastia, Amir H. Gandjbakhche

**Affiliations:** ^1^ Department of Computational and Data SciencesGeorge Mason UniversityFairfaxVAUSA; ^2^ National Institute of Child Health and Human DevelopmentNational Institutes of HealthBethesdaMDUSA; ^3^ Department of NeurologyCenter for Neuroscience and Regenerative MedicineUniformed ServicesBethesdaMDUSA; ^4^ National Institute of Mental HealthNational Institutes of HealthyBethesdaMDUSA; ^5^ Institut Langevin ESPCI‐ParisTechParisFrance

**Keywords:** classification, feature selection, machine learning, near‐infrared spectroscopy, traumatic brain injury, time series feature extraction, wrapper method

## Abstract

**Background:**

We have explored the potential prefrontal hemodynamic biomarkers to characterize subjects with Traumatic Brain Injury (TBI) by employing the multivariate machine learning approach and introducing a novel task‐related hemodynamic response detection followed by a heuristic search for optimum set of hemodynamic features. To achieve this goal, the hemodynamic response from a group of 31 healthy controls and 30 chronic TBI subjects were recorded as they performed a complexity task.

**Methods:**

To determine the optimum hemodynamic features, we considered 11 features and their combinations in characterizing TBI subjects. We investigated the significance of the features by utilizing a machine learning classification algorithm to score all the possible combinations of features according to their predictive power.

**Results and Conclusions:**

The identified optimum feature elements resulted in classification accuracy, sensitivity, and specificity of 85%, 85%, and 84%, respectively. Classification improvement was achieved for TBI subject classification through feature combination. It signified the major advantage of the multivariate analysis over the commonly used univariate analysis suggesting that the features that are individually irrelevant in characterizing the data may become relevant when used in combination. We also conducted a spatio‐temporal classification to identify regions within the prefrontal cortex (PFC) that contribute in distinguishing between TBI and healthy subjects. As expected, Brodmann areas (BA) 10 within the PFC were isolated as the region that healthy subjects (unlike subjects with TBI), showed major hemodynamic activity in response to the High Complexity task. Overall, our results indicate that identified temporal and spatio‐temporal features from PFC's hemodynamic activity are promising biomarkers in classifying subjects with TBI.

## 1 Introduction

Executive function involves various complex cognitive processes, such as solving novel problems, generating strategies or sequencing complex actions (Elliott, 2003). Executive dysfunction in subjects with Traumatic Brain Injury (TBI) has been reported in (Gioia & Isquith, 2004; McDonald, Flashman, & Saykin, 2002) and is believed to be related to a dysfunctional prefrontal cortex (PFC) or disruption in the connection of the frontal lobes and other parts of the brain (McDonald et al., 2002). Poor performance within the PFC of TBI patients, independent of frontal parenchymal lesions, has been reported by researchers (Cazalis et al., 2006; Langfitt et al., 1986; Levin, 1982; Vilkki, 1992).

Advancement made in functional neuroimaging provide tools needed for the sensitive assessment of functional abnormalities following TBI in various brain regions, including the PFC. In particular, functional magnetic resonance imaging (fMRI) of the blood‐oxygen‐level‐dependent (BOLD) signal (Heeger & Ress, 2002), which depicts blood oxygenation changes followed by localized neuronal activity, has been widely used to characterize the spatio‐temporal pattern of brain regional activity in individuals with TBI (McAllister et al., 1999) (McAllister et al., 2001) (Cazalis et al., 2006; Scheibel et al., 2003).

Although fMRI has traditionally been the modality of choice to study brain function of individuals with TBI, it is relatively expensive, and is permanently sited (Amyot et al., 2012). Less expensive and more portable functional neuroimaging modalities such as functional near‐infrared spectroscopy (fNIRS) (Villringer and Chance 1997; Amyot et al., 2012; Bunce et al. 2013) have been utilized less to study brain function of individuals with TBI. Similar to its fMRI counterpart, fNIRS is capable of capturing local hemodynamic changes over the execution of a functional task. However, compared to fMRI, fNIRS offers lower spatial resolution and provides higher temporal resolution. fNIRS measures continuous change in chromophores in the blood, by sending near‐infrared‐range light (usually of 700–1000 nm wavelength) through light‐emitters and detect the diffused reflecting light after interacting with brain tissue by the detectors that are placed a few centimeters away from the emitters. Oxygenated hemoglobin (HbO) and deoxygenated hemoglobin (HbR) are the targeted chromophores measured by fNIRS. HbO and HbR signals are formed through successive measurements made over a time interval of an experiment.

There are only a few studies that have utilized fNIRS to evaluate cerebral oxygenation and blood volume alterations during the execution of functional tasks in patients after TBI (Bhambhani, Maikala, Farag, & Rowland, 2006; Hibino et al., 2013; Merzagora, Izzetoglu, Onaral, & Schultheis, 2014; Merzagora, Schultheis, Onaral, & Izzetoglu, 2011). These studies have employed very small sample sizes, various cognitive stimuli, and different analytical techniques (Bhambhani et al., 2006) used fNIRS to investigate cerebral hemodynamic alterations in the prefrontal cortex in 25 subjects with TBI and 13 healthy control subjects while they performed the handgrip contractions task. It was reported that subjects with TBI demonstrated a significantly lower increase in oxygenation in both left and right dorsolateral prefrontal cortex (DLPFC; Bhambhani et al., 2006). Merzagora et al. (2011) examined the differences in the prefrontal hemodynamic activity of 5 TBI subjects and 11 healthy controls and reported significant lower mean HbO values for the subjects with TBI in comparison to healthy control subjects while performing an attenion‐based task, and suggested that fNIRS could be used to monitor the rehabilitation procedure for the patients with TBI (Merzagora et al., 2011). Hibino et al., (2013) conducted a study on 9 TBI and 47 healthy subjects to investigate differences between the two populations by analyzing the HbO changes captured from frontal to temporal cortices in response to 9 different cognitive rehabilitation tasks. They documented higher HbO changes for TBI compared to healthy control patients in the medial frontal region and higher left frontal HbO changes were reported for healthy controls in majority of the cognitive tasks (Hibino et al., 2013). Merzagora et al. (2014) investigated fNIRS to understand working memory subcomponents for 6 TBI and 11 healthy controls and compared the maximum hemodynamic response between the two populations. It was reported that TBI subjects' largest hemodynamic response was significantly higher than the healthy control subjects while performing a working memory task, in particular in the left DPFC. Overall, significant hemodynamic response differences between TBI and healthy control in the PFC (or its subcomponents) have been reported in all the studies discussed above.

The common methodology in the majority of the aforementioned studies (except for the work by Merzagora et al. (2014)) for comparing the hemodynamic responses between the two populations is the univariate statistical analysis where a single feature from the hemodynamic signal is utilized to investigate the difference between the TBI and healthy subjects by conducting a statistical testing. Although this is a valid approach to study the differences between TBI and healthy control populations, it does not fully exploit the potential hemodynamic features that may act as TBI's functional biomarkers. The approach of single‐ hemodynamic feature analysis, while capable of signifying a difference between the TBI and healthy subjects, is incapable of providing a general model to classify a new (unseen) subject to the TBI or healthy population. A complementary approach to the traditional methodology is the multivariate machine learning techniques (Bishop, 2006). In particular, supervised ML methods have been vastly used to differentiate task‐specific or resting‐state brain activity in brain–‐computer interface (BCI; Lotte, Congedo, Lécuyer, Lamarche, & Arnaldi, 2007) applications and infrequently to classify healthy subjects from individuals with a neurological disorders (Ahmadlou, Adeli, & Adeli, 2010; Bosl, Tierney, Tager‐Flusberg, & Nelson, 2011; Rizk‐Jackson et al., 2011; Stahl, Pickles, Elsabbagh, Johnson, & Team, 2012; Woon, Cichocki, Vialatte, & Musha, 2007). In the context of TBI studies using hemodynamic response, multivariate machine learning techniques can provide a measure of ranking different hemodynamic features according to their contribution in distinguishing TBI from healthy subjects and also enables classification of subjects according to their hemodynamic features. Conversely, feature combination through a machine learning classification method in which TBI and healthy subjects are characterized by multidimensional feature sets enables exploration of potential biomarkers in combination with each other. In this approach, the goal is to identify the feature space in which TBI and healthy subjects are characterized with maximum intrapopulation similarity and minimum interpopulation similarity. Various heuristic feature extraction techniques attempting to construct single or multi‐dimensional feature spaces from the hemodynamic signal to classify brain activity for the Brain–Computer Interface (BCI) applications have been proposed (Coyle, Ward, Markham, & McDarby, 2004; Fazli et al., 2012; Hai, Cuong, Khoa, & Van Toi, 2013; Holper & Wolf, 2011; Luu & Chau, 2009; Naito, Michioka, Ozawa, Kiguchi, & Kanazawa, 2007; Power, Falk, & Chau, 2010; Power, Kushki, & Chau, 2011; Sitaram et al., 2007; Stangl, Bauernfeind, Kurzmann, Scherer, & Neuper, 2013). However, few studies have attempted to identify the most efficient set of features from subjects of a population with purportedly distinctive brain activity to provide a unique characterization for the population (here TBI population). Selecting the optimum feature elements from a set of hemodynamic features is not a trivial problem. For instance, it has been shown that single features that may seem irrelevant in a single feature analysis can prove relevant in combination with other features (Domingos, 2012). Therefore, the full inherent biomarkers of the TBI subjects' hemodynamic signal may be determined by identifying the set of features that optimally characterizes the population.

The objective of this study is to identify the potential prefrontal hemodynamic TBI biomarkers that contribute in characterizing TBI subjects at the individual level through a multivariate feature selection technique. Employing the multivariate analysis instead of the commonly used univariate analysis was motivated by the findings in (Guyon & Elisseeff, 2003) that the features that are individually irrelevant in characterizing the classes may become relevant when used in combination. Furthermore, the multivariate machine learning techniques are sensitive to spatial distribution and subtle effects in the brain that would be undetectable using the univariate group analysis methods as the focus in these methods is on gross differences at group level. To identify the potential hemodynamic TBI biomarkers, hemodynamic response from a group of healthy and chronic TBI subjects while performing an event‐related complexity task is captured. We propose a procedure to identify only the trials with elicited hemodynamic responses and reject the trials with artifactual hemodynamic responses by imposing certain restrictions on the HbO and HbR signals. The average HbO and HbR signal are obtained by averaging the remaining trials. For every subject, a set of hemodynamic features from the average trials is obtained. The optimum set of functional biomarkers is obtained by employing the wrapper feature subset selection method (Guyon & Elisseeff, 2003) from the extracted features. Wrapper feature selection method utilizes machine learning classification algorithm to score different subsets of the hemodynamic features with respect to their predictive power. Finally, the accuracy of the identified biomarkers in characterizing the TBI population is evaluated by employing different classification techniques.

## 2 Methods

### 2.1 Participants

Seventy subjects participated in the two IRB‐approved studies (NCT01797549 and NIH 07N0139) from which data from nine subjects were not used in this analysis. Data from these subjects were excluded from the study either due to the problems in data collection or major motion or detector artifacts. Details of the procedure to identify subjects with major artifactual data are explained in the preprocessing section. Final number of subjects available for analysis was 61, 31 healthy controls (17 male and 14 female) and 30 TBI subjects (24 male and six female). Table 1 illustrates the demographic for all the TBI and healthy participants in this study.

**Table 1:** Demographic and clinical characteristics of the study population

	TBI (*n *= 30)	HC (*n *= 31)
Age (years), mean ± STD	37.8 ± 11.6	30.8 ± 8.06
Gender, % male	80.0	58.06
Education (years)	15	17.2
Time since TBI (months), median, IQR	21.5, 13–41	
Road traffic incident, %	50	
LOC >30 min, %	40	
Days in ICU, median ± IQR	3, 1–8	
Received Rehabilitation, %	43	

TBI, traumatic brain injury.

All TBI subjects fulfilled inclusion/exclusion criteria of NCT01797549, “Detection of Hemodynamic Changes in TBI Population With Functional Near Infrared Spectroscopy”, adults between 18 and 55 years of age who suffered a moderate or severe TBI, by DoD criteria, that is a head injury associated with Glasgow Coma Score between 3 and 12, loss of consciousness >30 min, alteration of consciousness or post‐traumatic amnesia >24 hr, or TBI‐related abnormality on neuroimaging (CT or MRI). All except one (examined at 2 months after injury) were tested over 6 months after injury with the median interval from TBI to NIRS testing as listed in Table 1. Twenty‐four TBI subjects (80%) reported persistent TBI symptoms by DSM‐IV criteria for post‐concussive symptoms, including cognitive symptoms (full neuropsychological testing data was not collected); six (20%) of the 30 were asymptomatic and fully recovered from their TBI at the time of NIRS testing. Twenty‐six (86%) TBI subjects are right‐handed, and 4 (14%) left‐handed, consistent with norms.

Thirteen TBI subjects received either inpatient or outpatient TBI rehabilitation after their acute hospitalization for their injury. The type and duration of their therapy reflected the severity of their injury and symptoms during the subacute period after their injury. Only 1 TBI subject was still receiving rehabilitative services at the time of enrollment in the study, a 28‐year‐old who suffered a severe TBI 8.5 years prior to enrollment in NCT01797549.

### 2.2 Experimental design

An event‐related paradigm in which subjects are required to evaluate the complexity of certain daily life activities was chosen for this study. The paradigm was originally designed and implemented in an fMRI experiment by Krueger et al. (2009) and has been shown to engage the PFC. Complexity task is selected for this study as it is known to engage the executive function that is impaired in the subjects with TBI (Paré, Rabin, Fogel, & Pépin, 2009; Stuss, van Reekum, & Murphy, 2000).

**Figure 1:**
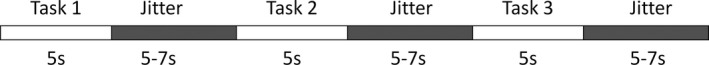
Experimental paradigm for the functional near infrared spectroscopy (fNIRS) data collection. Every trial lasted 5 s and was separated by a randomly assigned jittered interstimulus interval of varied interval of 5–7 s

In this paradigm, subjects were exposed to two classes of conditions: An experimental condition or the Complexity task and a control condition or the Font task. Stimulus presentation was controlled by the E‐prime software package (Psychology Software Tools, Inc., http://www.pstnet.com/eprime.cfm). Participants were first trained with a separate set of stimuli to familiarize them with the experiment. At the beginning of each trial, instructions describing the type of task (Complexity or Font) and the name of a daily‐life activity, for example, “stirring a cup of coffee” was displayed on a computer monitor for 4 s. For the complexity task, participants were asked to make a binary decision as to whether the activity name displayed corresponded to an activity with low complexity, for example, “stirring a cup of coffee” or an activity with high complexity, for example, “planning a wedding”, using a two‐button response pad. For the Font task, participants were asked to decide whether the instructions and the activity name shown represented the same or different fonts. Participants were prompted to respond as quickly and accurately as possible. A trial lasted for 5 s as the stimulus was presented for 4 s and was followed by 1 s after the stimulus disappeared. Trials were separated by a randomly assigned jittered interstimulus interval of varied interval of 5–7 s. 33 Font and 66 Complexity trials were randomly arranged within a 15‐min period of fNIRS data collection. Figure 1 visualizes the experimental paradigm and the corresponding durations of the tasks.

**Figure 2:**
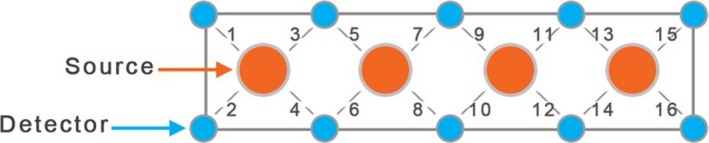
The functional near‐infrared spectroscopy (fNIRS) channel scheme. It is composed of 4 sources and 10 detectors, which form 16 source/detector pairs separated by 2.5 cm. The sensor pad is positioned on the subject's forehead

The hemodynamic response changes were recorded with a continuous wave fNIRS device with four light sources and 10 detectors (fNIR Devices LLC, Potomac, MD, USA). The distance between each source/detector pair was 2.5 cm. The configuration of probes for this device is denoted in Fig. 2. The lights were emitted from each source at two different wavelengths of 730 nm and 850 nm. The light sources were activated in sequence for collecting measurements from 16 different channels that spanned the forehead at 2 Hz.

### 2.3 Preprocessing

The raw intensity data measured at two wavelengths was normalized for all channel sites to compute the relative change by dividing each value of the intensity signal by the mean of the baseline signal. The intensity‐normalized data was then used to calculate the change in optical density (delta‐optical density). Delta‐optical density was computed for each wavelength as the negative logarithm of normalized intensity. For every subject, a differential pathlength factor (DPF) was calculated as the variable of age and the wavelength following the formula obtained in (Scholkmann & Wolf, 2013). Using the DPF values, the delta‐optical density was converted to changes in HbO and HbR, using the modified Beer–Lambert law (Delpy et al., 1988). HbO and HbR signals were low‐pass‐filtered using butterworth IIR frequency filter of order 10 with a cut‐off frequency 0.1 Hz. The filtered data was then detrended using the piecewise linear detrending to remove linear trends in the data. Trials corresponding to the High complexity stimulus were considered for this study. Trials were extracted by considering 11 s post‐stimulus onset. We considered 3 s after the trial ends, as it has been shown that a full hemodynamic change occurs over a 10–12 s period, after the stimulus is presented (Izzetoglu et al., 2005). To decrease the effect of motion artifacts or major detector artifacts during the data collection and also increase the relevance of the hemodynamic response to the presented stimulus, certain restrictions were imposed on the extracted trials. In an elicited trial, decrease in HbR signal is expected to be accompanied by an increase in HbO signal in the activated area (Plichta et al., 2006). Therefore, corresponding to every stimulus only trials in which HbO and HbR are negatively correlated were considered. Furthermore, to guarantee that the selected trials encompass brain hemodynamic activity elicited by the presented stimulus, trials in which HbO signal was (on average) larger than HbR were considered (see Results section). Finally, trials with negative HbO values were discarded from the analysis. HbO and HbR data corresponding to the High complexity trials were then block averaged across the remaining trials for every channel.

### 2.4 Feature extraction

As previously stated, the majority of the TBI fNIRS studies have attempted to construct a small feature space (feature spaces with 1 or 2 elements) to investigate the difference in the TBI and healthy populations. We address this issue by extracting 11 time‐ and frequency‐domain features and investigating their potential to be employed for characterizing the TBI and healthy subjects. We identify the optimum feature space for distinguishing TBI and healthy subjects by employing a features selection method in the next section.

Typically, the average HbO signal obtained in response to an eliciting stimulus embodies a positive deflection representing the activation in the channel that we refer to as the activity curve. The activity curve is the curve embodied in the HbO signal that is formed by an increase in oxygenation and its return to the same level of oxygenation. Depending on the nature of the features, they are extracted from the entire average HbO or the activity curve as follows:

1. Mean value of the HbO signal (HM),
2. Variance of the HbO signal (HV),
3. Left slope of the activity curve (CSL),
4. Right slope of the activity curve (CSR)
5. Kurtosis value of the HbO signal (HK),
6. Skewness value of the HbO signal (HS),
7. Area under the activity curve (CA),
8. Full width half maximum of the activity curve (CF),
9. Peak amplitude of the activity curve (CP),
10. Activity start time (CAS),
11. Discrete Fourier Transform (DFT) Coefficients of the HbO signal (HDFT)

**Figure 3:**
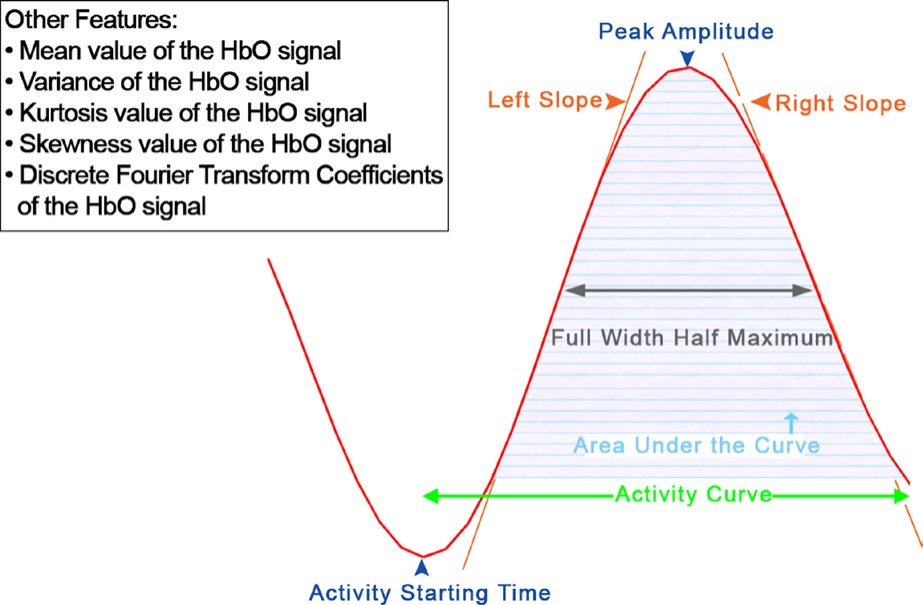
Visualizing the HbO signal (in red), activity curve and a number of hemodynamic features extracted in this study. The activity curve is a positive deflection representing the activation embodied in the HbO signal. The activity curve is formed by oxygenation's increase and its returns to same level of oxygenation

Figure 3 visualizes the HbO signal and the extracted features. Two slope values for the slope features. Left slope is computed between the points corresponding to the peak of the activity curve and where the activity curve starts and the right slope is computed between the points corresponding to the peak of the activity curve and where the activity ends. Furthermore, DFT provides a projection for the HbO signal with *N* data points in the time domain into the frequency domain by the following:

(1) $$c_{n}=\sum_{k=0}^{N-1}\text{HbO}\left(k\right)\exp\left(\frac{-2\pi ikn}{N}\right),\;n=0,\ldots N-1,$$

where *c*_n_ coefficients are a sequence of complex numbers that represents the amplitudes and shifts of a decomposition of the signal into sinusoid functions. HbO(*k*) is the value of the HbO time series at time *k*. Keeping a few coefficients and discarding the rest that provides a rough sketch for the original HbO signal is a common time series feature extraction technique (Mörchen, 2003). For this study, we kept the three coefficients corresponding to the very low‐frequency oscillations (VFLO) and low‐frequency oscillations (LFOs) ranging from 0.01 to 0.1 Hz. The VFLO and LFOs from the cerebral hemodynamics are shown to be associated with the spontaneous response and functional stimulation of the brain, respectively (Obrig et al., 2000). Furthermore, this range of frequency is known to be related to the cerebral autoregulation (Anderson et al., 2014; Chernomordik et al., 2016; Kainerstorfer, Sassaroli, Hallacoglu, Pierro, & Fantini, 2014; Liu et al., 2015) which is the specific intrinsic ability to maintain constant cerebral blood flow over a range of blood pressure and is known to be disturbed or absent in 49–87% of patients with TBI (Rangel‐Castilla, Gasco, Nauta, Okonkwo, & Robertson, 2008). All the other features result in one single value and are as follows:

Mean (HM): average signal value.

Variance (HV): measure of HbO signal spread. The variance formula for HbO signal with *n* data points as follows:

(2) $$\text{HV}=\frac{1}{n}\sum_{i=1}^{n}{\left({\text{HbO}}_{i}-\text{HM}\right)}^{2},$$

where HM is the average of the HbO signal and HbO_*i*_ is the *i*th data point of the HbO signal.

Skewness (HS): measure of the asymmetry of signal values around its mean relative to a normal distribution. If the HbO signal is symmetrically distributed, then HS will be 0.

Kurtosis (HK): measure of the degree of peakedness of a distribution of signal values relative to a normal distribution.

Activity start time (CAS): represents the time instant after stimulus onset when the oxygenation value in the HbO starts to increase toward its peak's amplitude.

Full Width at Half Maximum (FWHM): is commonly used to measure the width of a peak on a curve. As is illustrated in Fig. 3, the FWHM (“CF” in this study) value is obtained by computing the distance between points on the curve at which the values is half of the activity curve's amplitude.

### 2.5 Feature selection and pattern classification

To determine the optimal feature set (optimal combination of the aforementioned hemodynamic features) that enables distinguishing TBI subjects from the healthy subjects with the highest accuracy, we employed the wrapper feature selection method (Guyon & Elisseeff, 2003). Wrapper utilizes the machine learning classifier (popular predictors include decision trees, linear discriminant analysis, support vector machines and etc.) as a black box to rank different subsets of the features according to their predictive power. Wrapper addresses the problem of variable selection effectively in comparison to other techniques, as it is independent from the selected predictor and it can search the space of all feature combinations. To employ the wrapper method, one needs to define the classifier, a method of evaluating the performance of the predictor, and method of searching the feature space (Guyon & Elisseeff, 2003). In the wrapper method, a feature set is fed to the classifier and its performance is scored and the feature set with the highest rank, is selected as the optimal feature set.

In this study, due to the relatively small size of the feature space, an exhaustive search in the set of all the feature combinations was performed and for every possible combination of the feature sets a classification experiment was run. The Decision Tree (Breiman, Friedman, Stone, & Olshen, 1984) was utilized as the classifiers to evaluate different feature sets; 70% of the subjects (from TBI and health populations) were randomly selected for training purposes and the rest considered for testing purposes.

### 2.6 Classification evaluation

The TBI group was labeled as the positive class and the healthy group as the negative class. Generally, to assess the classification performance, evaluation indices are developed based on counting the number of TP, TN, FP, FN where,

1. True Positive (TP) – A subject belongs to the TBI population and is classified correctly as a TBI subject.
2. True Negative (TN) – A subject that belongs to the healthy population and is classified correctly as a healthy subject
3. False Positive (FP) – A subject that belongs to the healthy population and is classified incorrectly as a TBI subject.
4. False Negative (FN) – A subject that belongs to the TBI population and is classified incorrectly as a healthy subject.

Since the number of TBI and healthy subjects are comparable (33 TBI and 34 healthy subjects), the common metric of accuracy that weights TP and TN equally is appropriate for this classification problem (Satyasree & Murthy, 2013). The generalization performance of every classification experiment is assessed by random subsampling in which the process of randomly partitioning subjects into training and testing sets and executing the classification is repeated several times (1000 times in this study) and the average accuracy value of the 1000 classifications is considered as the overall classification evaluation index. For every classification experiment, the classification performance was evaluated and averaged across all the experiments. We report the average accuracy, specificity, and sensitivity which are the common metrics used for evaluating the classification performance for the class balanced problems (Tan, Steinbach, & Kumar, 2006). A dataset is class balanced if the classes are approximately equally represented. As it was discussed in the participant section, the dataset in this study is a balanced class dataset as it is composed (after the preprocessing) of 31 healthy and 30 TBI subjects.

The overall accuracy, specificity, and sensitivity values are determined by averaging the accuracy, specificity, and sensitivity values computed for every run of the random subsampling procedure.

Accuracy, specify, and sensitivity are computed as follows:

(3) $$\text{Accuracy}=\frac{\text{TP + TN}}{\text{TP + TN + FP + FN}}$$

(4) $$\text{Sensitivity}=\frac{\text{TP}}{\text{TP + FN}}$$

(5) $$\text{Specif}=\frac{\text{TN}}{\text{TN + FP}}$$

Sensitivity and specificity suggest how accurate the TBI and healthy subjects are detected through the classification procedure, respectively. In addition, once the optimum feature set is determined, we employ two more classification algorithms namely, Linear Discriminant Analysis (LDA; Welling, 2005), and Support Vector Machines (SVM; Suykens & Vandewalle, 1999) to evaluate their performance in distinguishing between the TBI and healthy subjects.

## 3 Results

### 3.1 Trial/Channel removal

**Figure 4:**
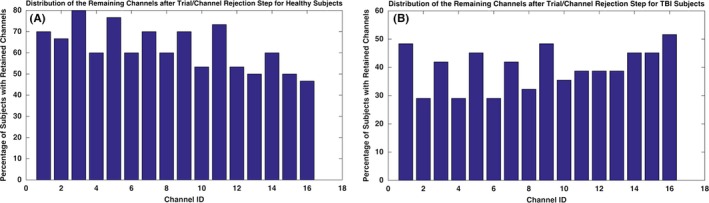
Channel distribution for the healthy and traumatic brain injury (TBI) populations after the channel/trial removal step is illustrated. For the TBI subjects, less number of subjects shares a common channel, whereas for majority of the healthy subjects share similar channels are kept. In the TBI population, more than half of the subjects share only channel 16. However, in healthy population except for channel 16, all the other channels are shared among more than half of the subjects

The three criterions discussed in the preprocessing section were applied on every single trial. A channel for which more than 80% or more of the trials were discarded was not considered for analysis. Subjects for which all the channel data were rejected were also discarded from the study. Six subjects (three TBI and three healthy subjects) were discarded from the analysis by applying the trial‐removal preprocessing step. The remaining channels for every subject contained only trials that were the most representative for hemodynamic activation in response to the High complexity task. Figure 4, illustrates the distribution of the retained channels across all the subjects after the trial/channel removal step. As it can be seen, the difference in the distribution of the retained channels between the two populations is clear. For the TBI subjects, a smaller number of subjects shared a common channel and the channel sites with elicited activity data were diffusely distributed. However, the majority of the healthy subjects shared similar channels. In particular, in the TBI population, more than half of the subjects shared only channel 16 and the rest of the channels were distributed among different subsets of subjects. However, in the healthy population except for channel 16, all other channels were shared among more than half of the subjects. The feature extraction and classification procedures in the sections below are performed merely on the channels for which the hemodynamic signal is kept.

### 3.2 Temporal feature extraction/classification

Temporal features were extracted from every channel of the subjects. For every subject, these features were averaged across all the channels to obtain the subjects' representative feature set. All the possible combinations of the generated features were considered for distinguishing the TBI from the healthy subjects using the Decision Tree classification algorithm. Table 2 illustrates the accuracy, specificity (accuracy of classifying healthy subjects correctly), and sensitivity (accuracy of classifying TBI subjects correctly) for the single‐feature classification. As it can be observed in the table, the largest accuracy is obtained for the feature space constructed by the left slope of the activity curve (CSL) variable with an accuracy of 65%. Poor classification performance was obtained for the remaining features. Although these variables seem to be irrelevant to the task of classifying TBI from the healthy subjects once used for single variable classification, they were not discarded in search for the optimal feature set. Findings in (Domingos, 2012) indicate that an irrelevant single variable in two class classification may be relevant once used in combination with other features. Therefore, we evaluated the classification performance among all the possible combinations of the generated features of different sizes (2047 potential feature sets for 11 features). Table 3 shows the classification performance for the optimum feature sets of different sizes. The optimum classification performance is obtained for the feature space constructed by the triple feature set of [CA, HDFT, CF] with the average classification accuracy of 85%. Sensitivity and specificity values computed for the corresponding classification suggest that TBI and healthy subjects are classified with accuracies of 85% and 84%, respectively. This finding suggests that on average, 26 TBI subjects (out of 30) and 26 healthy subjects (out of 31) are correctly identified for the feature space constructed by [CA, HDFT, CF]. It can also be observed in Table 3 that comparable classification performance is obtained for the optimum feature sets of size 4 and 5. Hence, it seems safe to conclude that the feature space constructed by these five hemodynamic elements (CA, HDFT, CF, CSL, and CSR) provide the most accurate distinction between the TBI and healthy subjects. Furthermore, a comparison between the sensitivity and specificity for the classifications with optimum feature sets (feature sets of size 3, 4, and 5) indicates that the TBI subjects are identified with marginally higher accuracy. The high sensitivity values signify the potential relevance of these five hemodynamic features to be used as biomarkers for subjects with TBI.

**Table 2:** Accuracy, specificity (accuracy of classifying healthy subjects correctly), and sensitivity (accuracy of classifying traumatic brain injury [TBI] subject correctly) of the classification experiments for the feature space constructed using one feature element. Accuracy, specificity, and sensitivity were computed by averaging their values over the 1000 classification experiments (random subsampling procedure). The largest accuracy value is obtained for the feature space constructed by the left slope of the activity curve (CSL) variable. Overall, the accuracy of correctly identifying the TBI subjects (sensitivity) is larger than the accuracy of correctly detecting the healthy subjects for feature set of any size

	Feature										
	HM	HV	HK	HS	CSL	CSR	CA	CF	CP	CAS	HDFT
Accuracy (%)	38 ± 9	57 ± 9	55 ± 9	55 ± 10	65 ± 10	57 ± 10	39 ± 10	57 ± 10	45 ± 10	58 ± 9	59 ± 10
Specificity (%)	38 ± 19	61 ± 18	56 ± 19	55 ± 19	61 ± 18	61 ± 19	39 ± 18	58 ± 18	42 ± 20	57 ± 16	58 ± 18
Sensitivity (%)	42 ± 19	55 ± 17	56 ± 17	60 ± 19	71 ± 18	54 ± 18	42 ± 18	55 ± 19	49 ± 19	62 ± 18	61 ± 18

**Table 3:** Classification measure obtained using the optimum feature sets of sizes 2–11 is presented. Accuracy, specificity, and sensitivity were computed by averaging their values over the 1000 classification experiments (random subsampling procedure). The optimum feature sets are selected from all the potential feature combinations of a certain size. Among all the combinations of features for a certain size, the one with the highest accuracy value is selected as the optimum feature set. The optimum classification performance is obtained for the feature space constructed by the triple of 3 features of “activity curve slopes (CS)”, “HbO kurtosis (HK)”, and “activity starting time (CAS)” resulted in the best separation between the traumatic brain injury (TBI) and healthy subjects. Comparison between the specificity and sensitivity indicates that in all the cases, sensitivity has been superior to the specificity meaning TBI subjects have been classified with higher accuracy

Size of the feature set combinations	Feature set with highest Accuracy	Accuracy (%)	Specificity (%)	Sensitivity (%)
2	[CA,HDFT]	81 ± 9	79 ± 15	82 ± 14
3	[CA,HDFT,CF]	85 ± 13	84 ± 16	85 ± 17
4	[CA,HDFT,CSL,CSR]	83 ± 14	83 ± 18	84 ± 18
5	[CA,HDFT,CSL,CSR,CF]	83 ± 14	83 ± 17	84 ± 18
6	[CA,HDFT,CSL,CSR,CF,CP]	78 ± 13	77 ± 18	80 ± 18
7	[HV,HS,HK,CA, CAS,CF,HDFT]	70 ± 13	67 ± 19	75 ± 18
8	[HV,HS,HK,CA, CAS,CF,HDFT,CSL]	70 ± 14	67 ± 20	74 ± 19
9	[HV,HS,HK,CA, CAS,CF,HDFT,CSL,CP]	67 ± 11	64 ± 19	70 ± 19
10	[HV,HS,HK,CA, CAS,CF,HDFT,CSL,CP,CSR]	67 ± 13	64 ± 19	71 ± 19
11	[HV,HS,HK,CA,CAS,CF,HDFT,CSL,CP,CSR,HM]	63 ± 11	60 ± 19	67 ± 17

**Figure 5:**
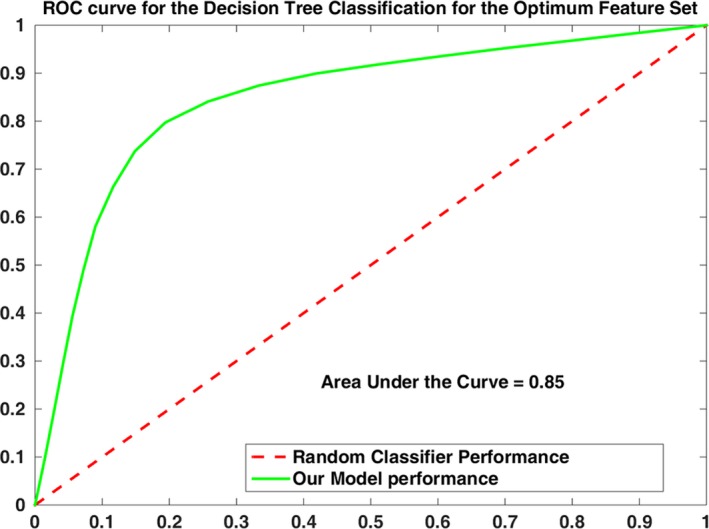
ROC curve for the classifying subjects into traumatic brain injury (TBI) and healthy groups, in the feature space constructed by the optimum feature set [CA, HDFT, CF]. Specificity and sensitivity values at each point of the graph are obtained by averaging the corresponding values across the 1000 run of the random subsampling procedure. Area under the curve of 0.85 is obtained for the constructed model, which signifies the high accuracy of the constructed classification model

In Fig. 5, Receiver Operating Characteristics (ROC) curve for the Decision Tree classifier in the feature space constructed by the optimum feature set is illustrated. An ROC curve illustrates the performance of the obtained classification model for the optimum feature set by visualizing the trade‐off between the sensitivity and the specificity. The area under the curve (AUC) quantifies the overall ability of the classifier to distinguish between the TBI and the healthy subjects. An ideal classifier has an AUC of 1 and a random classifier has an AUC of 0.5. Therefore, the larger the AUC, the better the performance of the classifier in separating the TBI subjects from the healthy subjects. Specificity and sensitivity values at each point of the graph are obtained by averaging the corresponding values across the 1000 run of the random subsampling procedure. The AUC of 0.85 obtained for the constructed model in the optimum feature space signifies the high accuracy for the classification model.

It is worth noting that the classification performance for the features of the optimal set: CA, HDFT, CF, CSL, and CSR in Table 2 suggests that 65% of accuracy (accuracy value obtained for CSL) is the optimal obtained performance if these features are used for the single variable classification. However, the feature space formed by combining these features improved the classification performance. In particular, presence of the variable “CA” in the optimal feature set verifies that variables with poor performance in separating the subjects for single feature classification can improve the classification if used in combination with other features. On the contrary, a few variables with relatively larger accuracy values (e.g., HV) for single feature classification are not improving the classification performance in combination with other features. These observations verify the significance of performing multi‐feature analysis.

For the optimal features set of [CA, HDFT, CF], we attempted to evaluate the performance of other commonly used classifiers and provide a comparison with the Decision Tree performance. In Table 4, the result of classifying TBI and healthy subjects in the feature space constructed by [CA, HDFT, CF] using the LDA and SVM (using the polynomial kernel) is illustrated. As results in Table 3 indicates, classification of TBI and healthy subjects using the Decision Tree algorithm outperforms the two other techniques.

**Table 4:** Classifying traumatic brain injury (TBI) and healthy subjects by characterizing subjects in the features space defined by the identified optimal feature set [CA,HDFT,CF] using three different classifiers. Decision Tree classifier outperformed LDA and SVM classifiers

Classifier	Accuracy (%)	Specificity (%)	Sensitivity (%)
Decision Tree	85 ± 13	84 ± 16	85 ± 17
Linear discriminant analysis (LDA)	64 ± 10	61 ± 17	72 ± 17
Support vector machine (SVM)	65 ± 9	55 ± 16	76 ± 14

### 3.3 Temporal feature extraction/classification without rejecting trials/channels

In this section, the efficacy of our proposed preprocessing step of imposing constraints on the selected trials is evaluated. As discussed in the Methods section, trial/channel rejection step is proposed to identify the trials with meaningful HbO and HbR. Therefore, to evaluate the efficacy of the proposed technique, we repeated the entire classification procedure without imposing any of the constraints that were introduced (see Methods section). This was done to reject artifactual trials and provide a comparison between the results of this process, which are shown in Table 2. However, to be able to provide a fair comparison, the six subjects that were discarded through the trial/channel rejection in the previous section, were not considered for the current classification experiments. Table 5, illustrates the optimal feature sets identified through the wrapper method (see Methods section) and the corresponding classification performance measures.

**Table 5:** Feature sets with the largest accuracy values were selected from all the potential feature combinations of different sizes. HbO and HbR signals have been averaged across all the trials without applying the trial/channel rejection procedure on the signals

Size of the feature set combinations	Feature set with highest accuracy value	Accuracy (%)	Specificity (%)	Sensitivity (%)
1	[CSR]	57 ± 10	51 ± 19	62 ± 20
2	[CSR,HS]	62 ± 11	58 ± 19	58 ± 19
3	[HM,HV,CA]	58 ± 13	52 ± 22	64 ± 20
4	[CP,HM,CSL,CF]	57 ± 11	54 ± 21	62 ± 18
5	[CP,HM,CSR,HK,CAS]	57 ± 12	57 ± 20	57 ± 19
6	[CP,HM,HV,CSL,CA,CF]	59 ± 14	57 ± 21	61 ± 19
7	[CP,HM,CSL,CSR,CAS,CA,CF]	58 ± 12	56 ± 18	61 ± 19
8	[CP,HM,CSL,CSR, HK,CAS,CA,CF]	55 ± 13	55 ± 19	57 ± 20
9	[CP,HM,HV,CSL,CSR,HK,CAS,CA,CF]	54 ± 12	54 ± 19	56 ± 11
10	[CP,HM,HV,CSL,CSR,HS,HK,CAS,CA,CF]	51 ± 10	49 ± 19	53 ± 19
11	[CP,HM,HV,CSL,CSR,HK,CAS,CA,CF,HDFT]	46 ± 10	44 ± 18	49 ± 19

Comparing classification performance in Tables 3 and 5 suggests that the trial/channel rejection has significantly improved the classification performance. This difference in the classification performance implies that trials in which HbO and HbR negatively correlated and average HbO is higher than average HbR contains hemodynamic response‐related to brain activation elicited by the stimulus.

### 3.4 Spatio‐temporal feature extraction

In addition to the temporal classification, a spatio‐temporal feature extraction and classification procedure was also considered to identify the features that enable distinguishing TBI subjects from healthy. In the spatio‐temporal classification, unlike the temporal classification approach, the extracted features for a subject were not averaged across all the channels. Therefore, for a single feature there were at most 16 different feature values (some of the channels may have been discarded from the study, see Methods section) associated to the different channels. The decision tree algorithm was employed to classify the subjects in this spatio‐temporal feature space constructed by considering the feature for every classification experiment. Decision tree seemed feasible for this classification task as the spatio‐temporal feature sets for the subjects contained missing values (for the discarded channels) and decision tree is known to be capable of handling the missing values (Safavian & Landgrebe, 1991). Table 6, tabulates the result of this approach, for all the extracted features.

**Table 6:** Accuracy, specificity (accuracy of classifying healthy subjects correctly), and sensitivity (accuracy of classifying traumatic brain injury [TBI] subject correctly) for the spatio‐temporal classification. Similar to the single feature temporal classification, HbO variance (HV) and activity curve's left slope (CSL) resulted in relatively larger classification accuracy. However, single variable spatio‐temporal classification outperformed single variable temporal classification. Similar to temporal classification, the accuracy of correctly identifying the TBI subjects (sensitivity) is consistently larger than the accuracy of correctly detecting the healthy subjects

	Feature									
	HM	HV	HK	HS	CSL	CSR	CA	CF	CP	CAS
Accuracy (%)	68 ± 11	70 ± 9	65 ± 13	72 ± 11	71 ± 10	68 ± 10	70 ± 10	65 ± 12	72 ± 10	65 ± 11
Specificity (%)	66 ± 18	68 ± 18	58 ± 21	71 ± 20	74 ± 117	67 ± 17	67 ± 18	65 ± 20	68 ± 17	61 ± 17
Sensitivity (%)	72 ± 18	73 ± 17	74 ± 18	75 ± 14	74 ± 18	70 ± 17	75 ± 17	66 ± 17	77 ± 16	72 ± 17

**Figure 6:**
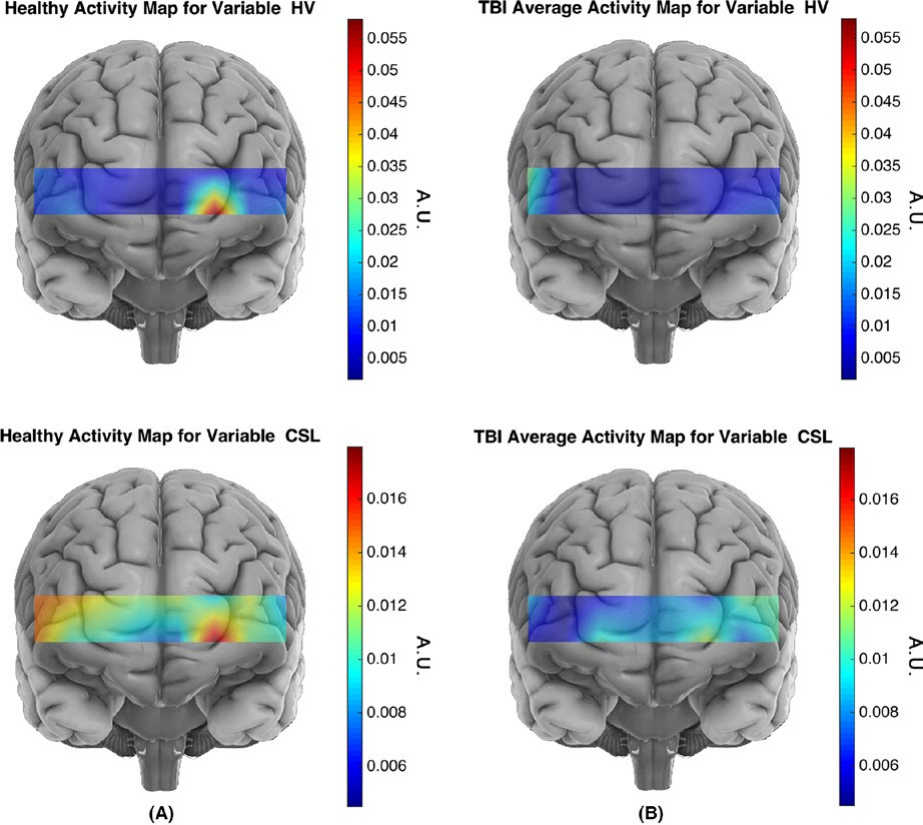
The average activity maps for the CSL and HV features for the healthy **(A)** and traumatic brain injury (TBI) **(B)** subjects are illustrated. The activity map for a spatio‐temporal feature associated to a population is obtained by averaging every subjects' (from the corresponding population) spatio‐temporal feature set. For the traumatic brain injury (TBI) population, the larger HV values are located at multiple locations with largest on the right hemisphere, whereas for the healthy population the largest HV is concentrated on the left hemisphere of the Brodmann area 10 (BA 10). Furthermore, healthy subjects on average show larger HV values for the HbO signal that indicates oxygenation signal has shown higher variation in the healthy subjects. The HbO signal in response to the High Complexity task for the healthy subjects shows larger variation and is spatially less diffuse than for the TBI subjects. Considering the activity map for healthy subjects, largest CSL values cover the left frontopolar of the BA 10. A comparison of healthy and TBI subjects' CSL activity map reveals that healthy subjects have shown larger CSL values in response to the High complexity task at all the sites of functional near‐infrared spectroscopy (fNIRS) data collection

As it can be observed in Table 6, the largest spatio‐temporal classification performances were obtained for the HS, CP, CSL, CA, and HV variables. Although, these classification experiments for the spatio‐temporal features do not provide significant distinction between TBI and healthy subjects, they outperform the obtained accuracy values for the corresponding single‐feature temporal classification (shown in Table 2). In Fig. 6, the average activity maps for the CSL, and HV for the healthy and TBI subjects are illustrated. The activity map for a spatio‐temporal feature associated to a population is obtained by averaging every channel's feature value across all the subjects (i.e., subjects from the corresponding population).

Distinctive spatial distribution for the HV feature is observed between the two populations. For the TBI population, the larger HV values are located at multiple locations with the largest in the right hemisphere, whereas for the healthy population the largest HV is concentrated in the left hemisphere within the Brodmann area 10 (BA 10; Ramnani & Owen, 2004). Furthermore, healthy subjects on average show a larger HV value for the HbO signal that indicates that the oxygenation signal has shown higher variation in the healthy subjects. Since, HbO signals are obtained from trials that indicate hemodynamic activation (see Methods section), it is safe to conclude that the HbO signal in response to the High Complexity task for the healthy subjects shows a larger variation and is spatially less diffuse than for the TBI subjects.

The left slope of the HbO signal's activity curve (defined as CSL in the Methods section) at a certain location describes the rate by which HbO's activity curve has started to increase toward its peak. Similar to the findings for the HV, the largest CSL values for the healthy subjects cover the left frontopolar area of BA 10.

### 3.5 Task load effects (i.e., parametric effects) in distinguishing traumatic brain injury from healthy population

As discussed in the Methods section, subjects in this study performed three loads of complexity task, Font, Low Complexity and High Complexity. In this section, we attempt to explore the parametric effects on the performance of the population classification procedure. To this end, the feature space from the identified optimal features was constructed for the Font and Low complexity tasks in a similar way it was constructed for the High complexity task. The Decision Tree algorithm was for classification as well. Table 7, illustrates the classification performance obtained for each task load. Results in Table 7 suggest that the as the task complexity decreases the classification performance also decreases. In other words, the difference between TBI and healthy subjects hemodynamic response is more prominent, while performing the task with higher loads.

**Table 7:** Comparison of the classification performance across tasks with different loads of complexity for the identified optimal feature set [CS, HK, CAS] using the Decision Tree classification

Task	Accuracy (%)	Specificity (%)	Sensitivity (%)
Font	52 ± 11	52 ± 18	53 ± 19
Low complexity	59 ± 10	58 ± 17	61 ± 18
High complexity	79 ± 13	74 ± 18	84 ± 16

## 4 Discussion

We attempted to identify the prefrontal hemodynamic biomarkers that provide the optimum distinction between the TBI and healthy subjects by a multivariate feature combination and temporal and spatio‐temporal classification approaches. To this end, we presented a novel approach for identifying single‐trial hemodynamic responses that encompass task‐related hemodynamic activity by imposing certain restrictions on a signal's statistical characteristic followed by a hemodynamic feature extraction procedure. To determine the optimum biomarkers from the extracted hemodynamic features, we investigated the effectiveness of the 11 extracted features from subjects' prefrontal hemodynamic response in separating TBI and healthy subjects. The extracted features were employed for two types of classifications, namely, temporal and spatio‐temporal classification.

In the temporal classification, the performance of 2047 classification experiments for every possible combination of features was evaluated. In every classification experiment, a distinct combination of the features was used to represent the subject's hemodynamic data. Optimum feature elements resulted in classification accuracy, sensitivity, and specificity of 85%, 85%, and 84%, respectively (Table 3). The sensitivity value of 85% obtained for the optimal classification experiment suggests that TBI subjects have been successfully characterized for the optimum feature set. Classification improvement that was achieved for the TBI subject classification through feature combination signifies the major advantage of employing multivariate analysis (as opposed to the univariate analysis) suggesting that the features that are individually or mutually irrelevant in characterizing the data may become relevant when used in combination.

For the spatio‐temporal classification, the performance of every single feature in distinguishing between the TBI and healthy subjects by incorporating the spatial characteristics to the feature set was evaluated. Optimum accuracy, sensitivity, and specificity of 72%, 75%, and 71%, respectively, were obtained for the spatio‐temporal classification (Table 6). The spatio‐temporal classification performance in comparison to the temporal classification was less significant. Less accurate performance of the spatio‐temporal classification may be explained by the fact that for every subject a number of channels may have been rejected and it causes the spatio‐temporal feature set to contain several missing values. However, for the temporal classification the average characteristics of the hemodynamic feature across the existing sites is considered and the missing data does not contribute in the classification procedure.

The selected optimum hemodynamic features set that effectively characterized TBI subjects with respect to their PFC hemodynamic response using the temporal classification are HbO's area under the curve (CA), HbO's, DFT coefficients of the HbO signal (HDFT), activity curve's full width half maximum (CF), activity curve's left slope (CSL), and activity curve's right slope (CSR). As discussed in the Methods section, HDFT is composed of four components of which two correspond to the magnitude of the very low frequencies that are associated to the spontaneous oscillations in cerebral oxygenation and the other 2 are the magnitude of low frequency between 0.07 and 0.1 Hz. The relationship of the magnitudes of these frequencies to the functional stimulus for the hemodynamic signal collected from the visual cortex has been investigated in (Obrig et al., 2000) and they are shown to be altered by the stimulation. The significance of these frequency magnitudes in our results, which are obtained in response to the High Complexity task is in line with findings in (Obrig et al., 2000) that suggests the relationship between these frequency magnitudes and functional stimulus and also claims that this functional response is observed over the PFC and is not bounded to the visual cortex. Furthermore, as discussed in the Methods section, the selected components of the HDFT feature are related to the cerebral autoregulation. The contribution of the HDFT components in separating TBI from healthy subjects is consistent with previous findings in which disturbance in the cerebral autoregulation in any degree of TBI has been reported (Rangel‐Castilla et al., 2008).

For the spatio‐temporal classification, HbO's skewness (HS), activity curve peak value (CP), activity curve's left slope (CSL), activity curve's area under the curve (CA), and HbO signal's variance HV were identified as the optimum feature elements. In Fig. 6, the spatial distribution of the HV and CSL variables for the healthy and TBI populations are visualized. The spatial distribution for HV and CSL signified the contribution of the left hemisphere of the BA 10 in separating the healthy subjects from the TBI for the spatio‐temporal classification. Healthy subjects showed a consistent pattern of engaging this region in response to the High Complexity task. This finding complies with the reports by Amyot et al. (2012), Krueger, et al. (2009) that BA 10 in healthy subjects is majorly activated in response to the High Complexity task. The HV's spatial distribution map for the TBI subject suggests that the TBI population have very low activation values across the entire PFC in comparison to the healthy subjects. This finding is in line with the previous study of (Sánchez‐Carrión et al., 2008) that reported patients with TBI show a pattern of cerebral hypoactivation in the right middle and superior frontal regions during working memory tasks.

We investigated the parametric effects of the task complexity in distinguishing the TBI and healthy subjects by employing the optimum feature set of the temporal classification for task with different loads. It should be noted that the complexity of the tasks (i.e., low or high complexity) had been determined in advance and was not dependent on subject's response. As it is shown in Table 7, the higher the task complexity, a greater distinction was obtained between the TBI and healthy subjects. This finding complies with a previous report for this specific functional task (Krueger, et al., 2009) in which distinct activation in the BA 10 for the High Complexity task was observed.

Overall, we successfully identified a set of hemodynamic biomarkers that enabled identifying and characterizing subjects with TBI from healthy subjects with a significant accuracy (sensitivity of 85% was reported in Table 3) through constructing a feature space that maximized the difference between TBI and healthy subjects. The reported accuracy value for the classification performance is the generalized accuracy that describes the likelihood of identifying a subject with TBI correctly, given its hemodynamic signals are characterized in the similar feature space.

As stated before, we intended to investigate the usefulness of the hemodynamic features in characterizing subjects with TBI. To this end, the hemodynamic features were extracted from HbO signals, whereas the hemodynamic features could potentially be extracted from other types of hemodynamic signals such as HbR or total hemoglobin (HbT). Furthermore, for certain features such as DFT, more number of features with employing 2N or 3N DFT could have been obtained that was not considered in this work. In our future studies, we will consider extracting hemodynamic features from other types of hemodynamic signals as well as more number of features with the purpose of creating larger feature space that can potentially improve classification of the TBI and healthy subjects.

It should be emphasized that although we showed that employing our proposed preprocessing step of trial/channel removal improves the classification performance, but it suffers from certain limitations and is susceptible of discarding valuable information. By applying this preprocessing step, channels for which 80% or more of the trials had been discarded were not considered for further analysis. This may result in discarding the majority of the trials from further analysis for certain channels. Furthermore, since certain channels are discarded from the analysis, the spatial distribution of the hemodynamic activity in the temporal classification can vary across subjects.

Finally, it is worth mentioning that our proposed approach of identifying TBI functional biomarkers using the fNIRS's hemodynamic signal has the potential to become a common approach in characterization of subjects with neurodegenerative, neurodevelopment disorders to further help clinical investigators to identify the underlying impairments of brain in the patient groups.

## Funding Information

We acknowledge the funding of the intramural program of the Eunice Kennedy Shriver National Institute of Child Health and Human Development, the National Institute of Neurological Disorder and Stroke, and Department of Defense in Center for Neuroscience and Regenerative Medicine.

## Conflict of Interest

None declared.
